# 

**Published:** 1823-11

**Authors:** 


### MONTHLY CATALOGUE OF MEDICAL BOOKS.

*** *It having been hinted to us that gentlemen, sending copies of their Works, prefer having their titles given at length, it is our intention, in future, to comply with this suggestion: but it is to be observed, that no books can be entered on this List except those sent to us for the purpose;—finding, in the lists hitherto transmitted, that the names of works have frequently been given as published, which have not appeared for weeks, or even months after.*

A Practical Treatise on Tropical Dysentery, more particularly as it occurs in the East Indies; illustrated by Cases, and Appearances on Dissection. To which are added, Practical Treatises on Scorbutic Dysentery, on the Morbus Chylopoieticus and Gastrodynia a Fame; with some Facts and Observations relative to Scurvy in general, and a short Account of the Scorbutic Disease that appeared at the Penitentiary, Millbank, Westminster. By R. W. BAMPFIELD, Esq. Surgeon; Author of an Essay on Hemeralopia, or Night-Blindness; formerly Surgeon of the Belliqueux and Warrior, his Majesty's Ships of the Line, serving in the East and West Indies; and now one of the Surgeons to the Royal Metropolitan Infirmary for Diseases of Children.—pp. 344. London, 1823.

A Treatise on Midwifery; developing new Principles, which tend materially to lessen the Sufferings of the Patient, and shorten the Duration of Labour. The second Edition, considerably improved, and illustrated with numerous Cases; comprising, also, additional Observations on premature Expulsion of the Ovum, and Retention of the Placenta. By JOHN POWER, M.D. Physician-Accoucheur to the New Westminster Lying-in Charity, and to the Dorcas Society; Member of the Royal Medical Society of Edinburgh; and Lecturer on Midwifery and the Diseases of Women and Children, &c. &c.—pp. 234. London, 1823.

A Course of Lectures on Chemical Science, as delivered at the Surrey Institution. By GOLDSWORTHY GURNEY.—pp. 310. London, 1823.

Mémoire sur quelques Découvertes récentes relatives aux Fonctions du Système Nerveux. Lu à Seance publique de l'Academie des Sciences, la 2 Juin, 1823. Par M. MAGENDIE, Membre de l'Institut et de l'Academie Royale de Médecine, &c.—pp. 25. A Paris.

Transactions of the Associated Apothecaries and Surgeon-Apothecaries of England and Wales.—pp. 424. London, 1823.

Observations and Commentaries on Medicine, compared, as a Science, with the other learned Professions, Theology, Law, &c.; together with comparative Illustrations, showing the important Advantages to be derived from the improved modern System of Medical Education and Practice, as now adopted in various parts of Europe and America. Also, an Elucidation of the Economy of the Human Body. The whole, not only intended as useful and necessary Information for the Student and young Practitioner, but for the general use and benefit of the Public. By ADAM DODS, M.D. formerly Surgeon in the Royal Navy; Author of an Essay on the Use and Abuse of Blood-letting, &c. &c.—pp. 82. Worcester, 1823.

Archiv für Medizinische Erfahrung im gebiete der Praktischen Medizin, Chirurgie, Geburtshülfe, und Staatsarzneikunde. Herausgegeben von den offentl. Lehrern des Heilkunde, Dr. HORN in Berlin, Dr. NASSE in Brun, Dr. HENKE in Erlangen, und Dr. WAGNER in Berlin. Jahrgang, 1823. Januar, Februar, Märtz, April.

Allgemeine Medizinische Annalen des Neunzehnten Jahrhunderts auf das Jahr 1823, oder; Kritische Annalen der Medizin als Wissenschaft und als Kunst vom dritten Jahrzehende des Neunzehnten Jahrhunderts an. Herausgegeben. Von D. J. FRIEDRICH PIERER, &c. und D. LUDWIG CHOULANT, &c. April, Mai, Junius, Julius.—Leipzig.

---

### NOTICE TO CORRESPONDENTS.

*Various Communications have been received, which, for want of room, we cannot specify.*

